# Address Delivered before the Chicago Gynæcological Society

**Published:** 1881-12

**Authors:** De Laskie Miller

**Affiliations:** President; Chicago


### CHICAGO MEDICAL

### JOURNAL & EXAMINER.

Vol. XLIII.—DECEMBER, 1881.—No. 6.

### Original Communications.

### Article I.

Address delivered before the Chicago Gynæcological Society, at the Annual Meeting, September 30, 1881, by DeLaskie Miller, M.D., President.

Gentlemen,—In obedience to the requirements of the rules which govern our society, I proceed to discharge one of the last duties devolving upon me for the current year.

In passing to the consideration of topics which I desire to present to you on this occasion, I do but reflect the sentiments of each member of the society, when I give expression to the feelings of sincere pleasure and devout thankfulness for the convalescence of one of our most esteemed brothers, who, more than a year ago, while in the enjoyment of robust health, and engaged in the active duties of the profession, was suddenly stricken down, and for a long time lay near the border line of the shadow land. We recall with pleasure the many virtues and the kindly disposition, the courteous bearing and the professional integrity which have ever characterized Dr. T. D. Fitch in his intercourse with members of the profession. While we deeply sympathize with our brother in his severe trial, we at the same time desire to express our thankfulness for his recovery and prospective restoration to usefulness in society, and to the enjoyment of social and domestic happiness for many years to come.

With your permission, gentlemen, I will devote a portion of the time at my disposal, to some impressions of the International Medical Congress of 1881.

On several accounts this congress was a remarkable one. First, on account of the numbers in attendance, which were greater than at any similar meeting of the profession which was ever convened. Second, the delegates represented a broader extent of the earth's surface than any other, including, as it did, representatives from every country where medicine is cultivated as a science. And third, among these delegates, moreover, were a large proportion of physicians who had made more than a local reputation. There were many whose names will ever be associated with the real and lasting improvements in medical science and art, which have been so numerous and so important during the time of the present generation.

The interest of the congress was not diminished by the fact that it was held under the patronage of Her Majesty the Queen, whose interest in every movement calculated to improve the race, and ameliorate suffering, is so well known throughout the civilized world. The proceedings were opened by his Royal Highness the Prince of Wales in person. The address of H. R. H. was a genuine surprise to most of those who composed the vast audience, for its pertinency to the occasion, and the admirable manner of its delivery—the Prince proving himself not only familiar, but also in hearty sympathy with the objects of the congress.

The opening address by the President, Sir James Paget, was an admirable production—comprehensive in scope, elevated in tone and dignity, presenting the aims and usefulness of the profession in a manner that stimulated every heart in that august assemblage with feelings of a just pride in our philanthropic profession.

I am sure I hazard nothing in asserting, that no meeting of the profession was ever held, ever since the world began, that would compare with this initial meeting of the Congress, in the effects produced upon the minds and emotions of the audience.

It would be inappropriate on this occasion to attempt, as it would be impossible to give even an epitome of the transactions of the different sections—fifteen in number—from personal observation. The proceedings will in due time be published, and will constitute a valuable addition to the medical literature of the current year.

To the section of obstetrics, Prof. Tarnier exhibited his latest improved obstetric forceps, modified in form from that first invented; he having found the instrument in its original form difficult to apply in certain positions of the head, and inefficient in action, except under favorable conditions. He claims for the instrument in its present form (minus the perineal curve), certain advantages over the ordinary double-curved forceps, which may be summarized as follows, viz.:

1st. Greater facility of application in certain difficult cases.

2d. A firmer grasp of the head.

3d. Permitting the movements of the head to take place in its passage through the pelvis, without the guiding aid of the accoucheur.

4th. Simplifying the application of force in extraction.

5th. That it is constructed on scientific principles.

Several eminent obstetricians commended the instrument as an improvement over the ordinary forceps. However, if I am not mistaken, the sentiment of the section, as expressed in the discussion, was decidedly averse to the claims of Prof. Tarnier. For it was contended, on the other hand, that even if the instrument of Prof. Tarnier was constructed on scientific principles, it was still imperfect, and liable to do injury in its use. It was asserted that greater difficulties would be encountered in its application than in the use of the ordinary forceps. Notwithstanding its vise-like grasp of the head, which is liable to injure by its continuous pressure, it is quite as liable to slip as the ordinary instrument. Loosening of the blades and relief of pressure at short intervals, are essential in forceps delivery. And again, the force in extraction should always be directed by the intelligence of the operator, as its direction should vary constantly as the head passes through the pelvis, and this change of direction is appreciable only by the information derived from the muscular sense while operating.

The subject of oöphorectomy was presented in two papers. One by the gentleman whose name has become synonymous with the operation—Battey's operation.

In the first paper, the object of the operation was claimed to be "The Artificial Induction of the Change of Life." This was assumed to be the pivotal principle that should govern the surgeon in determining the propriety of the operation in any given case.

It was asserted in the second paper, and confirmed in a statement of one gentleman in the discussion which followed, that menstruation sometimes continued after the operation had been performed. To explain this anomaly, one speaker claimed that menstruation was due, not to ovarian excitement, but to the influence of the Fallopian tubes! Are we justified, then, in accepting the philosophy upon which the operation is based if the function peculiar to the sex may continue unmodified after the operation?

It was claimed, moreover, by the advocates of the operation, that it was a remedy for insanity, for in two cases adduced, the patients became sane after the operation, and one gentleman suggested that this operation was destined to become recognized in state medicine, and that insane women should be subjected to the operation, to preclude the possibility of reproduction! On the other hand, however, cases were cited in which the patient became insane after the operation had been performed, in whom no symptoms of insanity had appeared before.

By some who professed to have had much experience, the operation was considered a curative means in severe hæmorrhage from myoma of the uterus. In the neuroses the result of the operation had been less satisfactory than in some other cases. The table of cases operated upon for this complication (hæmorrhage), which was circulated in the section, showed a mortality far greater than has ever occurred from the disease in the practice of any single physician who has treated such cases on the recognized principles applicable in such complications.

The operation was claimed by some who took part in the discussion, to be one of easy execution, even by the vagina, while another who had operated several times in this way, had relinquished this mode on account of its difficulty, and announced that hereafter he should operate by the supra pubic method. Again, one of the most distinguished ovariotomists stated that the operation was far from simple or of easy execution, being, in his opinion, more difficult than ovariotomy ; to which statement there seemed to be a general assent.

Again, it was more than hinted by some who had attained the highest eminence in the profession, that the operation had been resorted to with unjustifiable frequency, and not as a *dernier ressort*, as it always should be. All other means should be intelligently applied, and for a time sufficient to demonstrate their inefficiency, before deciding upon this operation. A practical hint of importance is contained in the fact, that in many cases in which the operation has been recommended, the evidence of great suffering is derived from the statement of the patient herself, and is mental or emotional, having no direct dependence upon the sexual organs.

Much difference of opinion was expressed upon the fatality of this surgical procedure. While the statistics presented by the author of the operation indicated a mortality as high as 18 per cent., it was claimed by others that the mortality should not exceed two, or at most, three per cent.

The conclusion reached by a majority of the section undoubtedly was, that the sphere of oöphorectomy and the indications for the operation, were not yet definitely settled.

Great interest was manifested in the subject. The best men in the profession participated in the discussion, with an apparent candor and freedom from prejudice that did honor to the character of the profession and this important section of the congress.

The papers upon the various subjects were grouped, so that all upon the same topic were presented consecutively. Thus upon displacements of the uterus and their treatment, several essays were read. For the most part the authors assumed that the consequences were due mainly to mechanical disturbances, or at least they would justify this inference ; for the one prominent idea appeared to be, that in the management of these cases, the mechanical reposition and retention of the organ was the one great indication. Two criticisms might be advanced upon these productions, viz.: 1st. It is assuming too much to affirm that in cases where displacement of the uterus is detected, that the symptoms and sufferings of the patient are invariably due alone to the dislocation of the organ. It is in accordance with the experience of every practitioner, that displacements are sometimes detected unexpectedly, and have only an incidental connection with the ailment for which treatment is sought. 2d. While mechanical treatment may be required, this alone will fill but imperfectly the indications of the case. These patients are, as a rule, anæmic. The functions of excretion and elimination are imperfectly performed, digestion and assimilation are perverted, and neurasthenia results. The manifest indications are, while we restore the organ to its normal position, we should adopt measures to remove local congestion and inflammation by suitable topical applications. We should excite the secretions and eliminate the effete materials from the system. We should improve digestion and assimilation, increase the quantity of a good quality of blood, and thus improve the tone of the tissues, without which all treatment will be nugatory.

The papers, and the discussion upon the operation for the partial or complete extirpation of the uterus, seemed to possess a peculiar fascination, occupying much time and exciting great interest. The gentlemen who took part in the discussion, however, were not always those who had enjoyed the best opportunities for observation. The *nonchalance* of the speakers would lead one to suppose that the operation was but slightly, if at all graver, than the extirpation of the tonsils. Could the sex, who are not themselves responsible for possessing this much-offending organ, hear but a tithe of the talk of removing it, I am sure they would regret, to their latest hour, the fate that had made them subject to such mutilation. Carcinoma was the disease for which this operation was recommended. The last speaker in the discussion closed by affirming that however early, well and thoroughly the extirpation might be performed, the patient, though she survived the operation, would surely die of the disease; to which sentiment there seemed to be a general assent.

One paper carried me back to the time of my earliest reading upon gynæcological subjects. It assumed that instead of laceration of the cervix uteri being the *fons et origo* of all uterine diseases, inflammation, with its consequences, ulceration, induration, *et omni genus* was the real pathology and the important condition to recognize, in the treatment of women, and that not a plastic operation, but local medication to remove these conditions, was the indication. Do the wheels of progress roll backward?

Two elaborate papers were presented upon post-partum hæmorrhage, which, after giving the causes and describing the conditions of this complication of labor, were devoted mainly to the management of this accident. The main reliance of one author in extreme cases (not new by the way) was the injection of a solution of perchloride of iron into the cavity of the uterus. This suggestion did not meet with the unchallenged approval of the section. In the discussion that followed, the great dangers liable to follow this mode of treatment were not ignored.

I was not a little surprised that the authors of these papers did not make more emphatic reference to two important means of management in sudden and great loss of blood after delivery, viz.: 1st. Direct pressure with the hand upon the site of the detached placenta, assisted by counter-pressure from without, sufficient to compress the uterus into a small compass, and down to the brim of the pelvis; and 2d, the importance of position, which can be utilized without interfering with other means in the least by raising the foot of the bed, so that, if need be, the body of the patient will form with the horizon an angle of 45°. This will secure the circulation of the little remaining blood in the system between the heart and brain, and thus bridge over the impending chasm of death, just ready to receive the patient.

But I would weary you, were I to follow this review further. It should not be inferred from the preceding, that no important or useful additions to the sum of our knowledge were made at the congress. I would by no means intimate that this was the case. On the contrary, it will only be necessary to remind you, gentlemen, that many of the best-known members of the profession throughout the world were present,—physicians who had made for themselves a world-wide reputation, and that they contributed their profoundest thoughts and the results of their large experience, thus adding to the interest of the meetings, and giving instruction to all who had the good fortune to be present.

Of the comparative interest or value of the work done in the different sections into which the congress was divided, it would be impossible to speak from personal observation. That irrelevant and crude remarks were occasionally made in the discussions is undoubtedly true; but these were not of sufficient frequency to give character to the proceedings of the meetings.

The addresses delivered at the general meetings were, without exception, full freighted with profoundest thought and advanced ideas sufficient to satisfy the exactions of the most inquiring minds of the day. That by Virchow was, as a plea for vivisection, classic; and when generally circulated, as it certainly should be, will exert a strong influence upon the popular mind now being arrayed against this practice for physiological and scientific ends. The following brief extracts are from the address. The animus of the objections are like the following:

"In the name of humanity, of morality, of religion, the suppression of experiment on animals is demanded. * * * The criterion is pain. Everything by which, in the way of experiment, pain is inflicted on the animal, is torture of animals, and so far immoral and contrary to religion." * * * The Professor answers: "The evidence that moral earnestness is failing in modern medical circles, is nowhere afforded. The reproach that Christianity is imperiled by vivisection, is worthy of Abdera. The assertion that the medical youth are inevitably "brutalized" by dissection and vivisection, is, as usual, snatched from the air; and it is also a calumny that vivisecting teachers have suffered injury to their morality."

That by Professor Pasteur, on the germ theory, excited great interest. It was from the experiments of Pasteur that Lister drew the inspiration which led to the valuable antiseptic method. And Dr. Davaine declares, "that his labors upon charbon (splenic fever or malignant pustule) had been suggested by Pasteur's studies on butyric fermentation, and the vibrion which is characteristic of it." In this address he gives the data of a further advance by utilizing these investigations, in the prevention of other transmissible diseases, promising to equal in importance that of vaccination as a preventive of variola.

The general address on surgery by Prof. Volkman, brought to view in clear light the recent improvements in his specialty. His own words shall give you an idea of his mode of treating his subject. "To-day we may say, with the deepest conviction, that the surgeon is responsible for every disturbance that occurs in a wound; that it is his fault if even the slightest reaction or redness is developed in it, or if an amputation is not healed by first intention. He must reproach himself severely if, after an operation, bagging of pus occurs, and especially if death occurs from pyæmia. He who cannot attain to this degree of perfection, may be converted from his former method of treatment to antiseptic methods like one who, having hitherto always prescribed senega for catarrh, now uses ipecacuanha. Of the storm that has swept over the fields of surgery during the past ten years he has not experienced a breath."

The masterly production of Huxley met to the fullest extent the highest expectations. In his sweeping survey of the progress of medicine from the earliest times to the present, it was possible to see, as with the natural eye, the dawn brightening into the effulgence of mid-day. Like some ship in mid-ocean surrounded by dense fogs which obscure the vision, so that the firmament, the horizon, and all objects, though near, are shut out from view, till the rays of the rising sun penetrate and dissipate the clouds, and render the atmosphere translucent, when every object is presented in distinct outline upon the "deep blue sea" even to the distant blending with the sky,—so the mists of ignorance and uncertainty, which have enveloped medicine and the race, have been gradually dissipated by the accumulated light of experiment and observation of the centuries. The Professor expressed the strongest confidence that medicine would ere long occupy a place with the exact sciences.

The social features of the congress added in no small degree to the interest of the occasion. The hospitality extended to the delegates, both public and private, was on a scale of magnificence which it is difficult to imagine could be excelled. The cordiality of the reception, and the personal attentions, were so spontaneous and constant as to excite the highest gratification in every stranger in attendance upon the congress. Though taxed to a great degree by the unprecedented attendance, nevertheless English hospitality was fully equal to the emergency.

I now pass to other topics of much interest to us as obstetricians. The study and the practice of obstetrics and gynæcology, at first view, appear to be distinct and independent departments of medicine. The difference, however, which in outward aspect are so marked, are in many respects apparent only. An interdependence and close relation exist in many, possibly in a majority, of the important cases which come under treatment. The complications and accidents of parturition, and the sequelæ of abortion, entail upon the sex many pathological conditions which are permanent, and cause great suffering and derangement of function. Nor is it stating the case too strongly to assert that these complications are for the most part avoidable. The inference that the accoucheur is largely responsible for much of the practice of the gynæcologist, is one that I do not propose to defend or deny.

Every obstetrician must have seen some cases, it may be not a few, in which serious consequences have resulted from injudicious interference, or from the neglect to render timely assistance. Unfortunately "meddlesome midwifery" is not always confined to the practice of the uneducated midwife. Like errors will be followed by similar results, though committed by the stronger sex.

I feel justified in presenting some suggestions upon topics, which for a long time I have considered important, viz.: How to prevent some of the accidents of labor.

Of course but little more than general statement is all that the time at my disposal will permit. Nor do I deem it expedient in this presence to elaborate in detail the propositions which I may advance.

At the present time great importance is attached to laceration of the cervix uteri. I do not introduce the subject on this occasion to discuss the pathological significance of this accident. Undoubtedly, however, minor cases of laceration have, not infrequently, been elevated into undue importance, and symptoms have been attributed to these slight cases which the history would not justify. Unfortunately this, like other important novel advances in medicine, has been carried to an extreme, which time, a more mature reflection, and careful observation will correct. My object is to consider some of the preventable causes of laceration of the cervix uteri.

This accident may result from the application of an undue amount of force, either natural or artificial, to the cervix, while the tissue of the os uteri is in a condition unfavorable for rapid dilatation. Who can doubt that the increasing frequency in the application of the obstetric forceps within the os uteri, is a cause of laceration of the cervix? To my mind, this practice, as a cause of the accident of which I am now speaking, is so undeniable as to require no argument to convince any. Demonstration could be adduced, not from experiment upon the cadaver only, but from observation of the effects upon the patient. I protest with emphasis against such use of the forceps, which has under the teaching of some, otherwise respectable authorities, become too general and too indiscriminate. Something should be left to the natural forces, in so important a function as parturition.

The undilatable os uteri may predispose to laceration of the cervix. Different conditions of the os may be met with, any one of which may constitute an important factor in retarding the process of dilatation; as irritability caused by the premature escape of the liquor amnii, rigidity from pre-existing disease, as chronic inflammation or hyperplasia. In such cases the contractions are liable to be violent. The indications to soften the tissue of the cervix, and to diminish the force of the contractions, can be fulfilled by applying emollients locally, and by the administration of sedatives, veratrum viride, morphia, chloroform, chloral, etc. By these means I am confident the dilatation has been facilitated, and laceration prevented. Again, not so infrequently as to be remarkable, have I met with cases of delay in the first stage of labor, from a cause which I suppose depended upon undue attachment of the membranes to the cervix uteri. In a model labor, the maturity of the ovum will be evidenced by a loosening of the membranes; the contractions of the uterus draw the os and inferior portion of the cervix away from the inferior segment of the membranes, while at the same time the membranes, with the liquor amnii, protrude through the os. By these means the os yields and dilates. If, however, the attachment of the membranes is too strong for this easy separation, labor is delayed, rupture of the membranes takes place before the os is dilated, and finally laceration takes place.

When delay in dilatation is met with, the tissue of the os and cervix being normal, the separation of the membranes from the cervix by the finger will be followed by the natural progress of the labor. I join issue with the assumption that the cervix is necessarily ruptured in labor.

*Vesico-vaginal fistula* may undoubtedly result from the unskillful use of instruments. More frequently, I believe, this accident should be attributed to some mismanagement of labor, or neglect of the patient after delivery. During a prolonged second stage of labor, the bladder should be emptied by the catheter, if necessary, at short intervals. After delivery, in severe labor, neglect to introduce the catheter at proper intervals will subject the weakened coats of the bladder to distension, and laceration into the vagina may take place. When any suspicion exists that these tissues have been injured by severe or long continued pressure, the catheter should be introduced at short intervals, not so much for the relief of suffering, as to prevent the laceration that would be liable to take place in the contused and weakened tissue of the bladder and vagina if distension should be allowed.

It will occur to every gentleman present, that great delay in the second stage of labor should not be permitted. Skillful delivery would certainly protect many patients from the distressing accident under consideration.

*Rupture of the perinœum* may result, like laceration of the cervix, from sudden distension of the tissues involved. A rapid termination of the stage of expulsion is far from being desirable. Time should be allowed for the tissues to yield. The length of time required mnst be determined by the attendant in any given case. But how gain time when the contractions are too violent for the safety of the perinæum? The effect of violent expulsive force may be lessened by directing the patient to cry out at the commencement of each contraction, and the advance of the presenting part should be retarded, as it can be by direct pressure. But what I believe to be more important in preventing rupture of the perinæum, is the judicious management of the head at the time of its expulsion, and this is accomplished when *extension* of the head is prevented, till the occiput has escaped in part beyond the arch of the pubis. If extension is allowed to take place too early, the effect would be to bring the largest diameter, viz., the occipito-mental, in relation with the vulva, so that a great increase of dilatation would be required in the escape of the head.

One so frequently hears practitioners recount the difficulties experienced in effecting the delivery of the placenta, that we would almost be justified in concluding that in this act nature is at fault, and that here we meet with a departure from the law that governs every other part of the great function of reproduction. Everywhere else we see in this function the most perfect and harmonious adaptation of means to an end, the parallel with which we will search for in vain elsewhere. Or may we not say rather, that the phenomena of expulsion of the placenta may not have been fully comprehended. It does not appear reasonable to suppose that, in this function, inherent difficulties should be met with often, so serious as to thwart the process of nature.

The ordinary delivery of the placenta is a very simple duty. At the same time, the proper delivery of the placenta is a very important duty. Nature requires but little assistance usually, but that little assistance should be given at the right time and in the proper manner. It is a serious error to forcibly extract the placenta too soon after the birth of the child. This practice causes hæmorrhage, syncope, and endangers inversion of the uterus. In natural labor, I would insist upon the necessity of permitting the contractions of the uterus to separate the placenta and close the sinuses. The time required for this would vary from fifteen minutes to half an hour. So much depends upon the manner of the delivery of the child, that it is impossible to do more than to approximate the time that would be required by nature to separate the placenta. Manifestly, the uterus would contract more slowly, and there would be a corresponding liability to hæmorrhage, were the child delivered suddenly, than if the inferior extremities were allowed to remain till uterine contractions expelled them.

Not only will the placenta be managed in this manner most successfully, but the patient will thus be best protected from post-partum hæmorrhage and septicæmia as well.

This word septicæmia leads me to add one other suggestion, and that refers to the necessity for the most careful cleanliness on the part of the accoucheur during the labor, and of the patient after delivery. There are other topics which I would be glad to present, but I have trespassed upon your patience too long already.

Of the work performed by this society during the year, it can hardly be necessary for me to speak except in general terms. The regular number of meetings have, I believe, been held, and I am happy to add that the members have faithfully responded to the requirements of our compact, in presenting papers and conducting discussions. The amount of work done by the society, as well as its quality, can best be appreciated by those who have been present at the meetings. The number of papers which have been printed has not been large, nor has the publication of the discussions been extended, and therefore it would be impossible from this source to form a just estimate of the quality of the work done or of the efficiency of the society.

I would do violence to my own feelings, did I neglect to thank the officers of the society for the punctuality and regularity with which they have, individually and collectively, discharged their several duties, and to each of the Fellows I extend my heartfelt thanks for their uniform courtesy during the year.

